# Seminal lipid profiling and antioxidant capacity: A species comparison

**DOI:** 10.1371/journal.pone.0264675

**Published:** 2022-03-08

**Authors:** Ulrike Jakop, Karin Müller, Peter Müller, Stefanie Neuhauser, Isabel Callealta Rodríguez, Sonja Grunewald, Jurgen Schiller, Kathrin M. Engel

**Affiliations:** 1 Leibniz Institute for Zoo and Wildlife Research, Berlin, Germany; 2 Institute for Reproduction of Farm Animals Schönow e. V., Bernau, Germany; 3 Department of Biology, Humboldt-Universität zu Berlin, Berlin, Germany; 4 Free University Berlin, Equine Clinic, Bad Saarow, Germany; 5 Faculty of Veterinary Sciences, University of Pretoria, Pretoria, South Africa; 6 Department of Dermatology, Training Center of the European Academy of Andrology, University of Leipzig, Leipzig, Germany; 7 Faculty of Medicine, Institute for Medical Physics and Biophysics, Leipzig University, Leipzig, Germany; University Hospital of Münster, GERMANY

## Abstract

On their way to the oocyte, sperm cells are subjected to oxidative stress, which may trigger the oxidation of phospholipids (PL). Applying MALDI-TOF MS, HPTLC and ESI-IT MS, we comparatively analyzed the PL compositions of semen and blood of species differing in their reproductive systems and types of nutrition (bull, boar, stallion, lion and man) with regard to the sensitivity to oxidation as well as the accumulation of harmful lyso-PL (LPL), transient products of lipid oxidation. In addition, the protective capacity of seminal fluid (SF) was also examined. The PL composition of erythrocytes and blood plasma is similar across the species, while pronounced differences exist for sperm and SF. Since the blood function is largely conserved across mammalian species, but the reproductive systems may vary in many aspects, the obtained results suggest that the PL composition is not determined by the type of nutrition, but by the relatedness of species and by functional requirements of cell membranes such as fluidity. Sperm motion and fertilization of oocytes require a rather flexible membrane, which is accomplished by significant moieties of unsaturated fatty acyl residues in sperm lipids of most species, but implies a higher risk of oxidation. Due to a high content of plasmalogens (alkenyl ether lipids), bull sperm are most susceptible to oxidation. Our data indicate that bull sperm possess the most effective protective power in SF. Obviously, a co-evolution of PL composition and protective mechanisms has occurred in semen and is related to the reproductive characteristics. Although the protective capacity in human SF seems well developed, we recorded the most pronounced individual contaminations with LPL in human semen. Probably, massive oxidative challenges related to lifestyle factors interfere with natural conditions.

## Introduction

The investigation of mammalian sperm lipids revealed large differences between animal taxa. This applies for the cholesterol/phospholipid ratio as well as for the type of phospholipids (PL). Although the occurrence of polyunsaturated fatty acyl (PUFA) residues, namely docosapentaenoic (C22:5) and docosahexaenoic (C22:6) residues, in the *sn*-2 position of the most prominent PL (glycerophosphorylcholine (GPC) and glycerophosphorylethanolamine (GPE)) is a common characteristic of all mammalian sperm studied so far, the type of the chemical linkage at the *sn*-1 position is variable. This means that sperm membrane lipids largely differ in their contents of diacyl, alkyl-acyl and alkenyl-acyl PL, which are referred to as ester lipids, ether lipids and plasmalogens as a particular group of ether lipids, respectively [1]. Boar and bull sperm possess mainly ether PL, which predominantly comprise plasmalogens in the bull [2]. In feline and human sperm, a mixture of ester and ether PL was detected and almost no plasmalogens were found [3, 4]. It remains unclear whether differences in the type of nutrition (herbi-, omni-, or carnivores) could provide a possible explanation for the observed PL patterns, or whether molecular and cellular interactions within the respective female reproductive system require particular PL arrangements in sperm membranes and, thus, a particular PL composition.

Lipids are the basic constituents of membranes, determine their physico-chemical properties and influence the organization and, thus, the function of embedded proteins (for review see [5, 6]). One important challenge to membrane lipids is oxidative stress, which is normally mediated by inflammatory cells such as neutrophilic granulocytes or macrophages [7]. In the male and female genital tracts, mediators of oxidative stress such as reactive oxygen species (ROS) are generated under pathological conditions, but also when the physiological immune response is triggered by semen after mating. Another source of ROS are the mitochondria of spermatozoa themselves, which produce the main energy for sperm homeostasis and motion [8–10]. Sperm-derived ROS may be released from defect or dead gametes.

Sperm are facing the dilemma that the fertilization process (i.e., the motion, acrosome reaction and successful fusion with the oocyte) requires an adequate level of membrane fluidity, which is normally ensured by the high amount of PUFA residues in membrane lipids [11–13]. However, a high extent of unsaturation indicates a high sensitivity towards oxidation. It must be noted that, because of their chemical properties, the alkenyl ether linkages of plasmalogens are particularly prone to oxidation – more than a "normal" double bond [14]. Oxidized fatty acyl residues are preferentially removed from the PL by the action of phospholipase A_2_ (PLA_2_) and an accumulation of LPL in sperm was assumed to be the result of lipid oxidation [15]. The formation of lyso-PC from unsaturated GPC was verified in experiments where isolated lipids with differences in the content of double bonds were artificially oxidized [16]. A recent investigation of human semen revealed that men with an extreme body mass index (BMI > 35) had a higher incidence to accumulate lyso-PC in sperm [17]. This is assumed to be a detrimental effect of metabolic disorders (such as the metabolic syndrome) which are accompanied by extensive oxidative stress [18].

In the present study, we analyzed the PL composition and the occurrence of LPL in sperm and SF from humans as well as from ungulates (pig, horse, bovine) and felids (lion) by MALDI-TOF, thin-layer chromatography (TLC) and electrospray ionization ion-trap (ESI-IT) MS. In contrast to gas chromatography (GC), complete lipid molecules are detectable with these methods and no previous saponification and derivatization are required. We hypothesized that the amount of LPL might serve as an indicator of oxidative stress in individual semen samples, since oxidation is accompanied by the hydrolytic cleavage of the unsaturated fatty acyl residue [15, 16]. The generated type and extent of LPL were also expected to depend on the species’ lipid composition (number of double bonds, plasmalogens), and a high level of LPL was assumed to correlate with poor sperm quality. To compare semen data with the situation in other cell types, we additionally investigated the lipid composition of erythrocytes and blood plasma (BP): it can be expected to be different if the PL composition is primarily related to cellular function. Lipid characteristics, which depend on nutrition, would be assumed to be similar in different cell types.

After release from testis and before ejaculation, sperm are matured and stored in the epididymis. During ejaculation, SF—amongst other functional molecules—provides enzymatic (e.g. superoxide dismutase, glutathione peroxidase) and non-enzymatic (e.g. vitamins, glutathione, taurine, albumins) antioxidants to sperm, which help protect the male gametes at their first step of their transit through the female genital tract [19, 20]. The distance from the site of sperm deposition to the site of fertilization in the oviduct and the time period required for sperm transit are traits of the respective reproductive system which may influence the extent of oxidative stress for the sperm. To assess the role of SF in sperm protection, we determined the overall capacity of the SF to scavenge free radicals, its protein content and its PLA_2_-hydrolytic activity in boar and stallion semen (cervical/intrauterine sperm deposition), bull and man semen (vaginal deposition) as well as lion semen ((pre)vaginal deposition). The comparative analysis of sperm lipids and their protection against oxidative stress will improve our understanding of adaptations in reproduction and helps to customize techniques of assisted reproduction according to the requirements of species and individuals.

## Materials and methods

### Chemicals

Chemicals, cell culture media and buffers, for sample preparation, solvents and the MALDI matrix 2,5-dihydroxybenzoic acid (DHB), were (unless stated otherwise) obtained in the highest commercially available purity from Sigma-Aldrich Chemie GmbH (Taufkirchen, Germany) and used as supplied.

### Sample collection

The semen samples were collected in local animal breeding centers during general semen production (bulls, boars, and stallions). Semen from captive bred African lions kept in two facilities in South Africa was collected during general anaesthesia. The experimental protocols were approved by the "Research Ethics and Scientific Committee of the National Zoological Gardens of South Africa" (NZG P12/17). Sample transfer to Germany was approved by CITES authorities (permits 125614, 138022). The use of human semen was approved by the ethical committee of Leipzig University, Saxony, Germany (No. 085/09-ff) and conducted in accordance with the ethical standard guidelines of the University under consideration of the Declaration of Helsinki. All patients signed a written informed consent form. Patient names were made anonymous by a numeric code.

### Preparation of spermatozoa and seminal fluid

#### Animal semen

Boar semen was collected by the gloved hand method, bull and stallion semen by use of an artificial vagina. In the case of lions, samples were collected during general anaesthesia by urethral catheterization [21]. The total volume of samples was recorded.

The motility of spermatozoa was assessed under a light microscope at 38°C. Sperm concentration and morphology were determined on fixed cells (0.5% formaldehyde in PBS). In the case of stallion sperm, the morphology was assessed after staining with Bromophenol blue and Nigrosine. To obtain SF, fresh ejaculates were centrifuged (700 × g, 5 min, 22°C) directly after ejaculation. An additional centrifugation step of the SF (12,000 × g, 3 min, 22°C) eliminated residual cells. Spermatozoa (pellet from the first centrifugation) were washed twice with HEPES-buffered solution (HBS, 150 mM NaCl, 5 mM HEPES, pH 7.4) by centrifugation at 700 × g for 5 min at room temperature and dilution of the pellet in HBS. SF and sperm were stored at -80°C until further analysis. In the case of lion semen, all spermatological analyses and sperm washing procedures were performed in South Africa. The samples for biochemical analyses (washed spermatozoa and SF) were frozen and transported to Germany in a dry shipper (cooled by liquid nitrogen).

#### Human semen

Human semen samples (n = 14) were obtained by masturbation from men of couples attending the fertility clinic at the Training Centre of the European Academy of Andrology at the University Hospital Leipzig. Men were 37.1 ± 6.9 years old, 1.74 ± 0.48 m tall, weighed 77.2 ± 13.8 kg and had a BMI of 25.4 ± 4.4 kg/m^2^. Smoking was reported by three, having a sauna by five donors. Three donors reported former cryptorchidism, four donors faced mumps during childhood. One donor reported high blood pressure. There is no information about the diet. However, due to the omnivorous character of humans, a mixed diet can be assumed.

For the determination of the sperm concentration, two independent dilutions of the homogeneously mixed ejaculate were prepared with 0.9% NaCl containing 5% 1 N HCl for sperm immobilization. For the motility assessment, two aliquots were investigated under a light microscope at 22°C. Sperm morphology was determined from two Papanicolaou-stained smears in accordance with the WHO lab handbook [22].

For the separation of spermatozoa from SF, a 1-ml aliquot of undiluted semen was centrifuged at 470 × g for 10 min. The supernatant was withdrawn, collected in a new 2-ml tube, and centrifuged at 4,500 × g for 10 min to pellet all cells. The SF was transferred into a 1.5-ml tube. The pellet was mixed carefully with 1 ml HBS, centrifuged at 370 × g for 10 min and the supernatant was discarded. SF and cell pellets were frozen at -80°C until further investigations.

### Preparation of erythrocytes and blood plasma

For animal blood samples, 1-ml aliquots were taken during routine health inspections. For lions, blood collection was performed under general anaesthesia. Blood was collected in EDTA-coated blood vials, carefully swivelled from side to side at least 10 times for mixing and stored at 4°C for a maximum of 12 h until further handling. To separate erythrocytes and BP, samples were centrifuged at 1,000 × g for 15 min. The upper layer (BP) was removed carefully. The intermediate buffy coat (with leucocytes) was discarded. The remaining pellet with the erythrocytes was washed twice in HBS as described above for spermatozoa. Erythrocytes and BP were stored at -80°C until further analysis. In the case of lion, washed erythrocytes and BP were transported to Germany in a dry shipper for biochemical analyses.

With humans, a 2.5-ml venous blood sample was collected in an EDTA-containing Monovette (Sarstedt, Nümbrecht, Germany) from each donor and processed as already described [23].

### Lipid extraction of spermatozoa, erythrocytes, seminal fluid and blood plasma

Lipid extraction was performed according to Bligh & Dyer [24] with slight modifications: 400 μL chloroform (CHCl_3_) and 400 μL methanol (CH_3_OH) were added to 20 to 100 × 10^6^ spermatozoa or erythrocytes in 400 μL HBS. Samples were vigorously mixed for 1 min. After centrifugation at 3,000 × g for 5 min, the lower organic phase was carefully collected by using a glass syringe. The upper aqueous phase was mixed again with 400 μL CHCl_3_. After a second centrifugation step, the organic phases were combined. Organic solvent was removed under a nitrogen stream, or by evaporation (Jouan centrifugal evaporator 1022, Thermo Scientific, Waltham, MA, USA).

Lipids of SF and BP were extracted as described above with the modification that 400 μL sample and 400 μL CH_3_OH were mixed with 800 μL CHCl_3_. Because lipid extraction from stallion SF was hampered by a significant protein content, proteins were precipitated by 400 μL CH_3_OH and centrifugation at 15,000 × g prior to lipid extraction.

### Electron spin resonance (ESR) spectroscopy

Electron spin resonance (ESR) spectroscopy can be applied to determine the capacity of body fluids to reduce radicals. SF is capable of eliminating free radicals due to reducing components. Spin-labeled (SL) fatty acids (FA) contain an unpaired electron and, therefore, mimic radicals in lipid molecules. The incubation of SL-FA and SF leads to a decrease in ESR signal intensity in dependence of the reductive capacity of SF.

The hydrolytic activity of SF can be determined by a SL-PL. The detached SL-FA from the PL backbone produces an isotropic signal in the ESR spectrum and the relative amount of hydrolyzed PL can be determined over time.

#### Analysis of the radical reduction capacity of seminal fluid by ESR spectroscopy

For measuring the radical reduction capacity of SF, the SL-FA 4-doxylpentanoic acid was used, which was synthesized as described in [25]. This SL-FA was dissolved in CHCl_3_/CH_3_OH (1:1, v/v) as a 1 mM stock solution. It was dried under a nitrogen flow and solubilized to a final concentration of 100 μM in HBS containing 1% BSA, which binds the SL-FA and, therefore, mediates its accessibility in aqueous solution. Twenty-five μL of the SL-FA solution were mixed with 5 μL of HBS and 20 μL of SF at 22°C and immediately filled into glass capillaries (Blaubrand^®^ intraMark Mikropipetten, 50 μL, BRAND GmbH & Co KG, Wertheim, Germany). ESR spectra were recorded at different time points over 30 min with the following parameters: modulation amplitude 2.5 G, power 20 mW, scan width 60 G, 1 × accumulated. From the spectra, the intensities of the mid field peaks were determined and related to those in the absence of SF. Kinetics of signal reduction were mono-exponentially fitted (SigmaPlot, Version 10.0, Systat Software Inc., San Jose, CA, USA) to determine the rate constant of the reduction. The latter was multiplied by the amount of the applied SL-FA to reveal the reduction capacity in SF per volume and also related to the sperm number in 20 μL of the original semen sample (sperm concentration in the ejaculate) to calculate the radical reduction capacity per sperm cell.

#### Analysis of lipid hydrolysis capacity of seminal fluid by ESR spectroscopy

The lipid hydrolytic activity of SF samples was measured by ESR using the PC analogue 1-palmitoyl-2-(4-doxyl-pentanoyl)-*sn*-glycero-3-PC with a SL-FA in *sn*-2 position, which was synthesized as described in [25]. This SL-PC was prepared and used as described in 2.6.1 as a 100 μM suspension in HBS containing 1% BSA. An aliquot of 25 μL of the SL-PC solution was mixed with 5 μL of of potassiumhexacyanoferrate (III) (K_3_[Fe(CN)_6_], 100 mM in HBS) to secure re-oxidation of reduced label, and 20 μL of SF at 23°C and immediately filled into glass capillaries. ESR spectra were recorded at different time points over 30 min with the above described parameters. The ratio of the isotropic signal in the ESR spectra from the produced free SL-FA was calculated via ESR spectra of known mixtures of SL-PC and SL-FA (without hydrolytic enzymes) as described in [26]. With this calculation, the hydrolytic activity of the SF can be determined over time and, further, related to volume and sperm number.

### Matrix-assisted laser desorption/ionization time of flight mass spectrometry (MALDI-TOF MS)

Total organic extracts of sperm, SF, erythrocytes and BP were investigated by MALDI-TOF MS. As MALDI matrix a 0.5 M 2,5-dihydroxybenzoic acid (DHB) solution in methanol [27] was used in all cases. All MALDI-TOF mass spectra were acquired on a Bruker Autoflex or a Bruker Auftoflex Speed mass spectrometer (both Bruker Daltonik GmbH, Bremen, Germany) utilizing a pulsed nitrogen laser and a Neodym-YAG laser emitting at 337 nm and at 355 nm, respectively. The extraction voltage was 20 kV and gated matrix suppression was applied to prevent the saturation of the detector by matrix ions [28]. For each mass spectrum, 200 single laser shots were averaged. The laser fluency was kept about 10% above threshold (i.e. the minimum laser fluency required to detect any signals) to obtain an optimum signal-to-noise ratio. In order to enhance the spectral resolution, all spectra were acquired in the reflector mode using delayed extraction conditions. Peak assignment was performed according to [3].

### High performance thin layer chromatography (HPTLC) and electrospray ionization ion trap mass spectrometry (ESI-IT MS)

Dried lipid extracts were dissolved in chloroform and automatically spotted onto a normal phase HPTLC glass plate (Merck KGaA, Darmstadt, Germany) by a Linomat device (CAMAG, Muttenz, Switzerland). Chloroform/ethanol/water/triethylamine (30:35:7:35, by vol.) was used as the mobile phase. After drying at room conditions for 15 min, the separated lipid fractions were visualized by dipping the entire plate into primuline (Direct Yellow 59, Sigma-Aldrich, Taufkirchen, Germany) solution (50 mg/l in acetone/water (80:20, by vol.)). The lipids in each spot were automatically eluted by a Plate Express™ TLC plate reader (Advion, Ithaca, NY, USA) with methanol as solvent and analyzed by direct transfer into the ESI-IT mass spectrometer.

ESI-IT MS was performed on an Amazon SL mass spectrometer (Bruker Daltonik GmbH) by direct infusion. Conditions were the following: spray voltage 4.5 kV, end plate offset 500 V, nebulizer gas 7 psi, drying gas (N_2_) 3 l/min, capillary temperature 180°C, flow rate 3 μL/min, sheath gas (He) flow rate 25 a.U. Spectra were recorded in the enhanced resolution mode by positive or negative ionization with a maximum ionization time of 50 ms.

### Statistical analysis

Statistical analyses were either performed using IBM SPSS Statistics 24 (SPSS Inc., IBM, Armonk, NY, USA), or GraphPad (version 8.4.0, GraphPad Software, San Diego, CA, USA). According to the low sample numbers, Spearman correlations were calculated between the different variables collected on individuals of a given species. Wilcoxon signed-rank test was used for comparison between the two groups in artificial LPC administration. The criterion for statistical significance was an error probability of < 0.05 in all tests.

Data were visualized as box plots including mean values (relative contents of PL). The boxes extend from the 25^th^ to the 75^th^ percentile. The line represents the median, the “+” the mean. Whiskers go from the minimum to the maximum. For the fitting of the hydrolysis curves Sigma Plot 14.0 (Systat Software, Inc.) was used.

## Results

### Semen characteristics

Semen characteristics of the investigated species are summarized in Table 1. Although the human samples met the WHO requirements in almost all characteristics, a poor morphology was recorded for about 3% of the normal sperm. In lions, most sperm with morphological defects had acrosome defects, midpiece defects, distal plasma droplets or looped tails, leaving about 25% of the sperm classified as morphologically normal. The motility was in a similar range in all species, with high individual differences in the lion.

**Table 1:** Ejaculate and sperm parameters of the investigated species.

	Boar	Stallion	Bull	Man	Lion
**N**	10	8	16	14	15
**Volume** [ml]	300 ± 82	56 ± 21	5.6 ± 2.1	3.0 ± 1.1	0.21 ± 0.17
**Sperm concentration** [10^6^/ml]	397 ± 189	232 ± 146	2087 ± 950	121 ± 92	1977 ± 1557
**Total motility** [%]	70 ± 8	68 ± 17	70 ± 7	58 ± 6	46 ± 29
**Morphology** [% normal sperm]	73 ± 13	62 ± 19	93 ± 8	3.1 ± 1.9	25 ± 18

Data are given as mean ± standard deviation.

### Lipid composition of semen and blood

**Fig 1:**
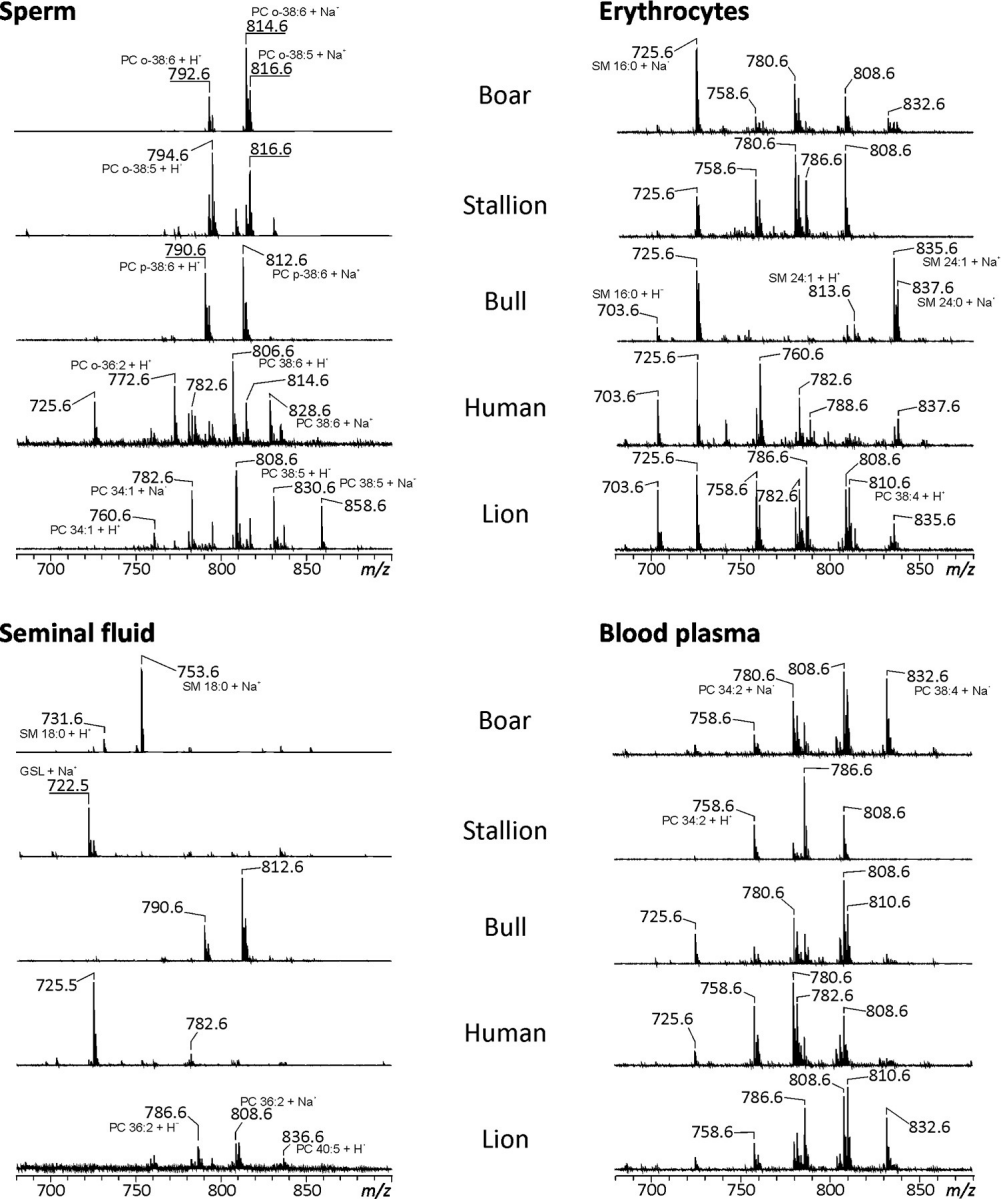
MALDI-TOF mass spectra of sperm, seminal fluid, erythrocytes and blood plasma samples. Extracted lipid-containing fractions from sperm, seminal fluid, erythrocytes and blood plasma samples of boar, stallion, bull, man and lion were mixed 1:1 with 0.5 M 2,5-dihydroxybenozoic acid (DHB) dissolved in methanol as matrix. A volume of about 0.75 μL of this mixture was applied onto an MTP 384 target plate ground steel BC (Bruker Daltonik GmbH, Bremen, Germany) and mass spectra were subsequently recorded in the positive ion mode on a Bruker Autoflex Speed mass spectrometer (Bruker Daltonik GmbH). The mass-to-charge (*m/z*) ratios of the most prominent peaks are assigned.

**Fig 2:**
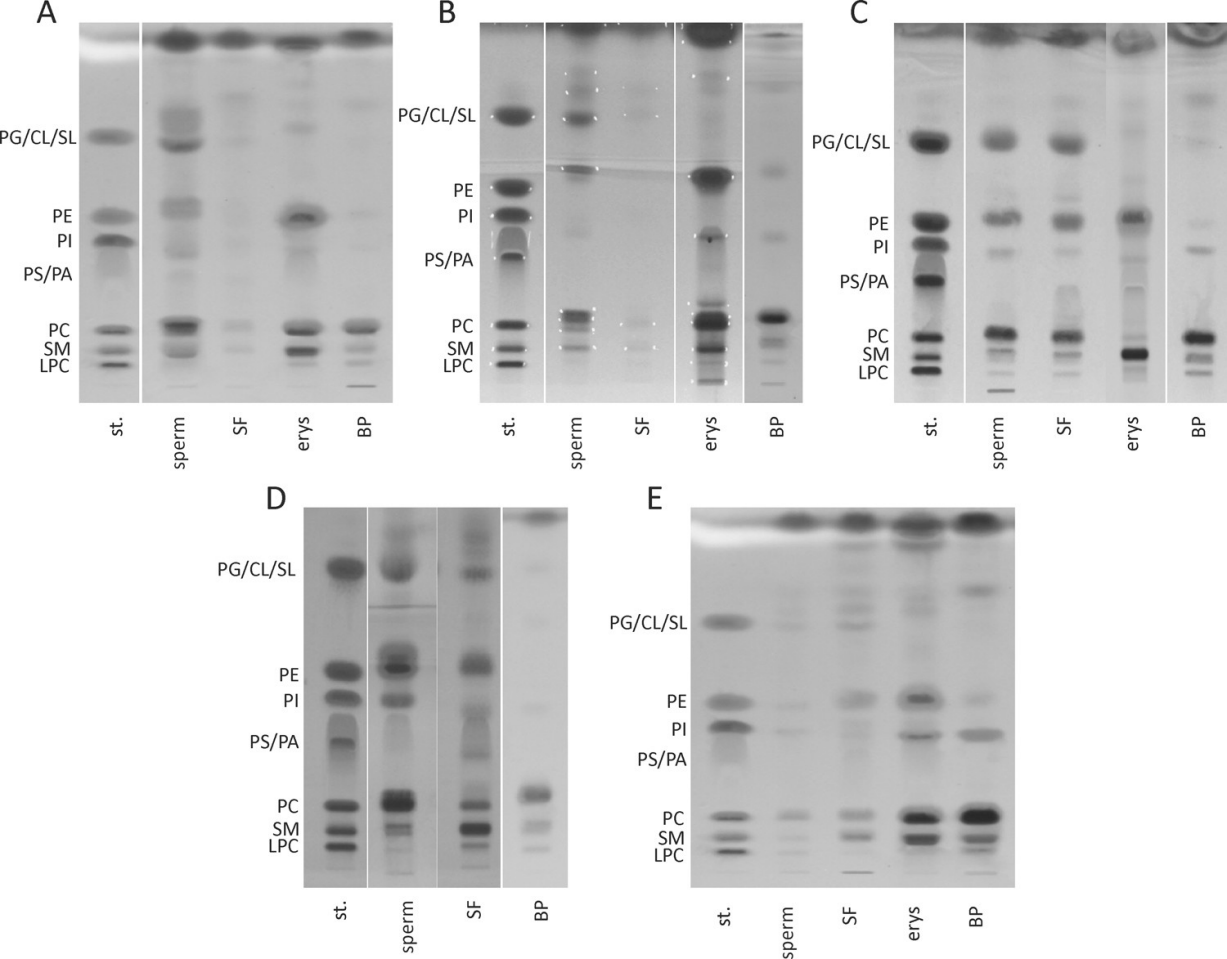
TLC-based separation of lipid-containing fractions of sperm, seminal fluid, erythrocytes and blood plasma samples. Extracted lipid-containing fractions from sperm, SF, erythrocytes and BP samples of boar (A), stallion (B), bull (C), man (D) and lion (E) were separated on normal phase HPTLC glass plates (Merck KGaA, Darmstadt, Germany) using chloroform/ethanol/water/triethylamine (30:35:7:35, by vol.) as the mobile phase. For visualization of the separated lipids, the plates were dipped into primuline solution (Direct Yellow 59, Sigma-Aldrich, Taufkirchen, Germany; 50 mg/l in acetone/water (80:20, by vol.)), dried under room conditions for 15 min and illuminated with UV light (366 nm). Lipid spots were marked with a pencil as demonstrated in B (white dots). For original TLC pictures see S9 Fig. BP–blood plasma, SF–seminal fluid, st–standard lipid mixture.

Positive ion MALDI-TOF MS analyses of organic extracts primarily detect GPC and sphingomyelins (SM) with high sensitivity. The GPC and SM fingerprint analyses of selected sperm, SF, erythrocytes and BP samples are shown in Fig 1. In addition, TLC separation of the crude lipid extracts was performed to obtain more detailed insights into the differences between all lipid classes (Fig 2). Finally, lipid fractions separated by TLC were directly analyzed by ESI-IT MS. Spectra of LPC, SM, GPC, GPI and GPE fractions are shown in S1–S6 Figs. It has to be noted that in some samples, not all PL classes were detectable (Fig 2). Some fractions separated into two distinct spots that were analyzed separately. According to the high volumes of boar (approx. 250 ml) and stallion SF (approx. 100 ml), the extracted volumes of SF (Fig 2A and 2B) contained much less PL compared to the other species, which is reflected by the poor spot intensities.

#### Interspecies differences of semen lipids

Considering spermatozoa, our former investigations were confirmed. Samples from boar, stallion and bull contain a limited number of GPC species (Fig 1 and S3 Fig), which almost exclusively comprise ether lipids with C22:6 or C22:5 residues linked to the *sn-*2 position of the glycerol backbone (corresponding to GPCo-16:0/22:6, GPCo-16:0/22:5 and GPCp-16:0/22:6) [3]. Only a small amount of ether-GPC in boar and stallion sperm, but most of the ether-GPC in bull sperm, can be classified as plasmalogens, i.e., are characterized by alkenyl linkages (corresponding to GPCp-16:0/22:6) [2]. Stallion sperm also showed GPC species with shorter chain lengths, such as PC14:0/14:0 and PC14:0/16:0 next to highly unsaturated acyl-acyl compounds and the predominant ether lipids. In lion and human sperm, a mixture of primarily ester- and some ether-GPC was observed with a moderate amount of ester-GPC containing more than four double bonds. Especially in lion sperm, only low amounts of highly unsaturated PC species were detected. The MALDI-TOF spectra of all sperm extracts (Fig 1) were dominated by GPC, solely in human sperm, a significant contribution of SM was visible (*m/z* 725.6 corresponding to SMd18:1/16:0 + Na^+^).

The lipid composition of SF differed throughout the species, but was rather simple. In boar and human SF, SM dominated, while the sodium adduct of a glycosphingolipid, namely the neutral glycosphingolipid Hex-Cer (d18:1/16:0), gave the most pronounced peaks in stallion SF. Regarding the GPC composition of the SF, only bull SF resembles the GPC pattern observed in the respective sperm. The lipid composition of lion SF is also similar in some part to that of the sperm and contains a mixture of ester- and ether-GPC.

**Fig 3:**
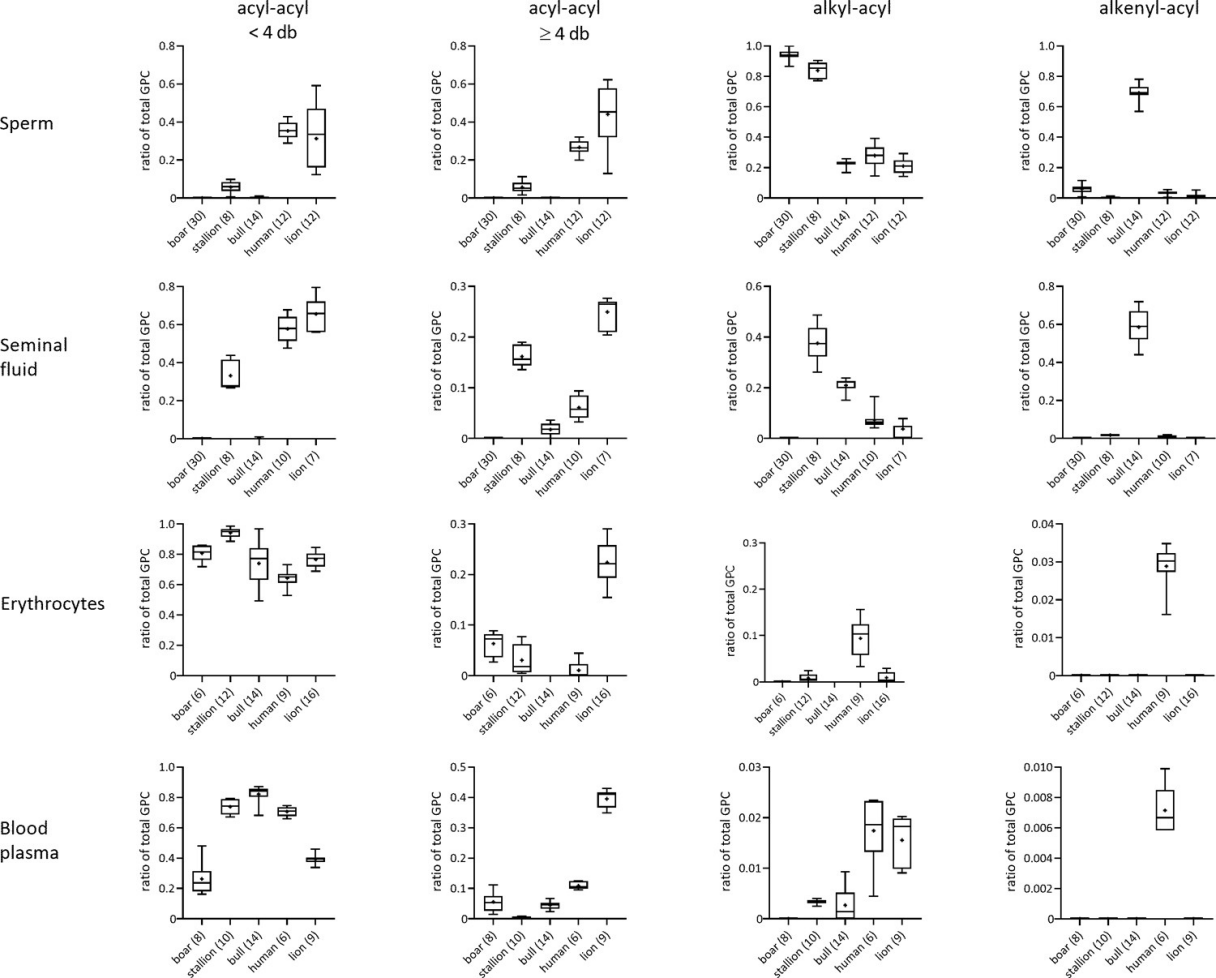
Relative amounts of glycerophosphatidylcholine (GPC) species in sperm, seminal fluid, erythrocytes and blood plasma samples. Extracted lipid-containing fractions from sperm, seminal fluid, erythrocytes and blood plasma samples of boar, stallion, bull, man and lion were investigated by MALDI-TOF mass spectrometry. Signal intensities of all GPC from one spectrum were summarized and set to 1. Relative amounts for the categories (I) GPC with two fatty acids bound to the *sn-*1 and the *sn-*2 position and less than four double bonds in total (*acyl-acyl <4db*), (II) GPC with two fatty acids bound to the *sn-*1 and the *sn-*2 position and more than or equal four double bonds in total (*acyl-acyl ≥4 db*), (III) GPC with an ether bond in position *sn-*1 to an alkyl group and a fatty acid bound to the *sn-*2 position (*alkyl-acyl*) and (IV) GPC with an ether bond in position *sn-*1 to an alkenyl group (plasmalogens, *alkenyl-acyl*) were calculated for each sample. The number of samples investigated is given in brackets after the species names. Data over all samples from one species are depicted as box plots with whiskers representing the smallest and the largest value. The line represents the median, the mean is represented by “+”.

The TLC pattern confirmed GPC as the most prominent PL class in sperm, followed by GPE and SM (Fig 2). In stallion sperm, GPC splits into three moieties representing the diacyl, alkyl-acyl and alkenyl-acyl species. The same applies for boar sperm, where GPC and GPE split into two moieties each. In accordance with the previous results, Fig 3 quantifies the relative abundance of ester- and ether-molecules of GPC as well as the degree of saturation of ester-GPC. In sperm and SF, it is obvious that the occurrence of ester-GPC is reciprocally related to the occurrence of ether-GPC across the animal species. In contrast to boar, stallion and bull sperm with almost exclusively unsaturated GPC, human and lion sperm contain about 40% more saturated ester-GPC (< 4 db).

The composition of the SM fraction in parts differed not only between sperm and SF, but also between species (S2 Fig, S2 Table). In lion sperm samples, almost exclusively SMd18:1/16:0 (sum formula SM34:1;O2, for shorthand notation see [29]) was detectable. In human sperm, the SM fraction split into two spots, the lower one consisting of SMd18:1/16:0 (see also Fig 1, MALDI-TOF MS), the upper one of long and very long-chained SM, namely SM with carbon chains from 24:1 to 30:4. The composition of the bull sperm SM fraction was very heterogeneous and contained many very-long chained even-numbered and unsaturated carbon chains, resulting in *m/z* 863.7 (SMd18:1/26:1), *m/z* 909.7 (SMd18:1/30:6), *m/z* 913.7 (SMd18:1/30:4), *m/z* 937.7 (SMd18:1/32:6) and *m/z* 965.7 (SMd18:1/34:6) for the sodium adduct [30]. The SM fractions of sperm and SF in boar additionally contained very-long SM (*m/z* 873.7, 901.7, 927.7 and 929.7), which might contain odd-numbered carbon chains representing the sodium adducts of SM45:4;O2, SM47:4;O2, SM49:5;O2 and SM49:4;O2. These were also present in stallion sperm, however, in lower concentrations. In cases where both residues are combined, no sufficient intensity was obtained in the MS/MS spectra to allow unequivocal peak assignments.

The GPI content was low in the majority of samples. Therefore, a direct analysis by ESI-MS was not possible in all cases. In cases where the analysis could be performed, the composition of the GPI fraction was quite simple, except for human sperm. GPI of human sperm consisted of short- and long-chained GPI species, as well as of saturated and highly unsaturated ones. In boar, bull and lion the fatty acyl chain lengths of GPI molecules were shorter in semen compared to blood samples (S4 Fig).

Regarding GPE, there were huge differences among the different samples and species (S5 and S6 Figs). Whereas in boar, bull, stallion and human sperm, the majority of the GPE were highly unsaturated and belonged to acyl-acyl, alkyl-acyl or alkenyl-acyl GPE, lion sperm consisted mainly of monounsaturated and low amounts of polyunsaturated acyl-acyl GPE. In SF, GPE species were mainly highly unsaturated and ether-linked in boar, bull and lion and monounsaturated in human SF.

A further predominant component of all sperm is cardiolipin (CL). This lipid class was also found in the SF of bulls and lions (Fig 2C and 2E).

#### Differences between semen and blood lipids

Both the MALDI-TOF and ESI-IT mass spectra of erythrocytes and BP reflect a higher diversity of GPC species, particularly if compared to the spectra from boar, stallion and bull sperm or SF. No similarity exists between erythrocytes and sperm as well as BP and SF from the same animal species. This particularly applies for the dominance of ether lipids including plasmalogens in boar and bull sperm. These lipids are only found in traces, or do not occur at all in erythrocytes or BP of these species. Regarding the animal species with sperm consisting of mainly ester-GPC (human, lion), the spectra of erythrocytes or BP in fact show some similarities, but are not at all congruent to the spectra of sperm or SF. Particularly lipids with highly unsaturated fatty acyl residues, which are characteristic for semen samples, are nearly completely missing in blood of all investigated species. Blood samples are more or less consistently dominated by moderately unsaturated ester-GPC (Fig 3). The largest contribution of unsaturated ester-GPC can be found in the blood samples from lion. Except for the human erythrocytes, ether-GPC is missing, or much less abundant. Plasmalogens are only present in blood samples from man, however, to a very low extent (Fig 3, lower right).

Erythrocytes and BP of all five species are characterized by huge amounts of PC and saturated SM and only small interspecies differences were observed. In contrast to the situation in semen, there is a similarity between the lipid species found in erythrocytes and BP of the same animal species. In bull erythrocytes, this becomes only detectable, if GPC spots are analyzed by ESI-IT MS after TLC separation, since the MALDI-TOF MS spectra of bovine erythrocytes are dominated by SM (Fig 2C). SM is detected in higher amounts in all erythrocytes, and is exclusively visible in bovine and human BP. Particularly in human BP (in the same manner as in human sperm), the SM fraction separated into two spots (Fig 2D). The lower consisted of saturated short-chained SM while the upper spot contained unsaturated long (>20 C) and very long-chained (>24 C) SM (S2 Fig). The SM fraction of erythrocytes was similar among the species and consisted mostly of SMd18:1/16:0, SMd18:1/24:0 and SMd18:1/24:1.

TLC analyses revealed that SM and GPE are much more abundant in erythrocytes than in sperm. Particularly, bull erythrocytes consist primarily of SM (in accordance with MALDI-TOF MS fingerprint) and GPE, but only very low amounts of GPC (Fig 2C). Comparing the lipid patterns of erythrocytes with that of BP, the latter contained only traces of GPE. Solely in the BP from bulls and lions, the significant occurrence of GPI was obvious and only minor in the other samples (S4 Fig). CL was not detected in blood. The ESI-IT MS revealed that erythrocytes of boar contained many mono- and diunsaturated acyl-acyl GPE species, which can already be seen from the localization of the spots on the TLC plate (Fig 2A), whereas erythrocytes from bull additionally consisted of alkyl-acyl GPE (S5 Fig). Those in stallion also contained only a few double bonds and were mainly of the ether type. In lion, erythrocytes GPE were highly unsaturated acyl-acyl and alkyl-acyl species. In BP samples from boar, bull, stallion and human, the GPE fraction consisted of saturated, mono-, di- and tri-unsaturated lipids.

### Accumulation of lyso-PL in semen and blood

Except for man and bull, the accumulation of LPL was minor in semen, generally not exceeding 5% of all GPC lipid species in single individuals (Table 2). Lyso-GPC (LPC) and formyl-LPC (fLPC) are distinguished, because LPC results from oxidation of ester-GPC, whereas fLPC typically stems from oxidized plasmalogens, where the alkenyl linkage can be oxidized under generation of an aldehyde [32]. In line with this, the largest moiety of fLPC was detected in bull semen due to its significant plasmalogen content. No correlation was found in any of the investigated species between the amount of (f)LPC in sperm and sperm motility or morphology. Amongst the investigated species, the highest individual moieties of LPC related to all other GPC species were observed in sperm (54%) and SF (87%) of individual men. SF comprises significantly higher amounts of LPL than sperm (fLPC in bulls, *P* = 0.007 and LPC in men, *P* = 0.003). In human, stallion and boar sperm samples subjected to TLC separation, LPC amounts were too low for a reliable detection by ESI-IT MS (Fig 2). However, in bull and lion sperm, the LPC composition is different, regarding the presence of higher unsaturated LPC species such as LPC18:3 and LPC22:6 in bull sperm.

**Table 2:** Radical reduction capacity of seminal fluid (SF), phospholipase A2 activity, protein concentration and occurrence of lyso-glycerophosphatidylcholine (LPC) and formyl-LPC (fLPC) in organic extracts of sperm and SF of different species.

	Boar	Stallion	Bull	Man	Lion
**Radical reduction capacity b** [10^−2^/min]	1.2 ± 0.4	0.0	4.6 ± 3.0	8.6 ± 5.9	1.1 ± 1.1
**Radical reduction capacity (normalized)** [pmol/(min*10^6^ sp.)]	4.2 ± 1.2	-	3.0 ± 2.4	106 ± 47	3.9 ± 5.9
**Lipid hydrolysis** [%/10 min]	1.6 ± 0.6	1.6 ± 0.3	90 ± 27	11.3 ± 4.8	5.4 ± 5.2
**Lipid hydrolysis (normalized)** [pmol/(10 min*10^6^ sp.)]	6.3 ± 4.4	11.6 ± 6.2	63 ± 24	203 ± 184	10.3 ± 13.2
**Protein concentration** [mg/ml]	45 ± 29	26 ± 17	91 ± 19	53 ± 11	17 ± 15
**Protein concentration (normalized)** [mg/10^6^ sp.]	0.15 ± 0.12	0.14 ± 0.13	0.06 ± 0.03	0.75 ± 0.60	0.05 ± 0.07
**LPC or fLPC in sperm** [% of all (PC+LPC+fLPC)]	in rare cases	in rare cases	**fLPC:** 5.2 ± 13.5	**LPC:** 9.9 ± 15.1	in rare cases
**LPC or fLPC in SF** [% of all (PC+LPC+fLPC)]	in rare cases	in rare cases	**fLPC:** 14.2 ± 10.4	**LPC:** 42.1 ± 21.4	in rare cases

Data are given as mean ± standard deviation. LPC and fLPC were related to the sum of intact glycerophosphatidylcholine (GPC), LPC and fLPC as calculated from the respective peaks in the MALDI-TOF positive ion spectra in samples from different individuals. Details for the measurement of the other parameters are given in the Materials and Methods section.

In contrast to sperm and SF, where noticeable amounts of LPL were only visible in individual samples from bulls and men, LPL could be found in erythrocytes and the BP from all species. Whereas the ESI-IT LPC mass spectra of erythrocytes from boar, bull and stallion are quite similar and display high amounts of LPC16:0 and LPC18:0 as well as minor amounts of LPC18:1, LPC18:2 and LPC18:3, LPC spectra from lion erythrocytes are mainly composed of LPC18:0 and only a small amount of LPC16:0 (S1 Fig). In all BP samples, the amounts of LPC18:1 and LPC18:2 were higher compared to erythrocytes. Among the individual species, BP differed regarding the amounts of LPC16:0 and LPC18:0. Additionally, LPC20:3 and LPC20:4 could be detected in BP of boar, bull and lion.

### Protective power of seminal fluid

The capacity of SF to scavenge free radicals differed strongly between the individual species (Table 2). This process was faster in SF of men compared to all other species. Normalizing the reduction rate (b-value) to the number of sperm in the respective aliquot of semen revealed that the radical reduction capacity was more than ten times more effective in human compared to the animal species. Note that human, stallion and lion sperm are smaller compared to bull and boar sperm [31], where a larger membrane area needs to be protected by SF. No radical reduction occurred in stallion SF. The rate constant of radical reduction was correlated to the sperm concentration in boar semen (r_s_ = 0.636; *P* = 0.048) and a similar tendency was calculated in human (r_s_ = 0.495; *P* = 0.072). There was no correlation to sperm quality in any of the remaining species. However, the radical reduction rate was negatively correlated to the relative amount of fLPC in bull semen (r_s_ = -0.572; *P* = 0.021). An extensive accumulation of LPL in sperm of all the investigated species only occurred in few of those individual samples where a low radical reduction capacity of the SF (normalized to sperm number) was observed.

The hydrolysis of PL was evident in the SF of all species. The by far fastest hydrolysis was determined in bull SF (Table 2). The hydrolysis over time differed considerably between the species, apparent by the different curve shapes (S7 Fig). The fitted curves followed a linear trend (boar, stallion), or an exponential growth to maximum trend (bull, lion, human). To compare the means of the individuals between the different species, the absolute value at the 10-min time point was used for the comparison—particularly to overcome the problem of the different curve shapes. Normalizing this hydrolysis rate at 10 min to the number of sperm revealed that human SF had a three times more effective hydrolytic capacity than bovine SF (Table 2). In bull semen, the rate of hydrolysis after 10 min is positively correlated to the moiety of sperm with normal morphology (r_s_ = 0.770; *P* < 0.001). However, the hydrolytic activity is not correlated to the amount of sperm LPL in any of the species.

The protein concentration in SF differs between the species as well. It is most pronounced in bull and lowest in lion. Related to the sperm numbers, human SF provides five to 15 times more protein to the sperm cells in an ejaculate than the SF from the animal species. The species with the highest sperm concentration (bull and lion) provide only one third of the amount of protein per sperm compared to boar and stallion. The protein concentration correlates with the rate constant of radical reduction in bull, man and lion (r_s_ = 0.544, 0.547, 0.681 and *P* = 0.029, 0.043, 0.010, respectively) and with the rate of hydrolysis in lion (r_s_ = 0.587; *P* = 0.045). This information is missing for boars, since the protein content was not determined in all relevant SF samples. The protein concentration was, however, negatively correlated to the sperm motility in man (r_s_ = -0.739; *P* = 0.003) and in lion (r_s_ = -0.655; *P* = 0.021). A negative correlation was also established between protein concentration and morphology in lion (r_s_ = -0.721; *P* = 0.005) and as a tendency in stallion (r_s_ = -0.683; *P* = 0.062).

## Discussion

### Relatedness of species is causative for the PL composition of sperm

With the results of the present study, previous studies on the GPC composition of boar, ruminant, human, and felid sperm [2, 4, 23, 32] could be confirmed and extended. The prevalence of ether-GPC (alkyl-acyl pattern) and the occurrence of plasmalogens, representing a special class of ether lipids with alkenyl-acyl pattern, in sperm of bull and boar were verified. In addition, ether-GPC were also found to be incorporated in stallion sperm, where a few short-chained GPC species with ester linkages were highly abundant as well. Therefore, the predominance of ether-GPC seems to be characteristic of ungulate sperm, whereas the incidence of plasmalogens is restricted to boar and specifically bull sperm. This can be explained by evolutionary aspects. Bovine and porcine species belong to the order of even-toed ungulates (Artiodactyla). Horses represent the order of odd-toed ungulates (Perissodactyla). Thus, this particular feature of the sperm lipid composition, which is similarly found for the lipid class of GPE, is rather determined by the close relatedness of species than by the type of nutrition, which is herbivorous in both, bovine and horse.

In line with this, sperm lipids of omnivorous species such as boar and human also differ profoundly in their composition. Even though pigs belong to the omnivorous species, the nutrition of breeding boars is mainly based on herbal food, while it can be assumed that men have eaten rather an omnivorous diet with a significant amount of meat. Therefore, diets for bulls, horses and boars mainly comprise unsaturated FA in comparison to the diets of men and particularly lions. Considering lion sperm, the high percentage of more saturated fatty acyl residues of mainly acyl-acyl GPC and GPE can indeed be related to the strict carnivorous nutrition. Nevertheless, a significant amount of unsaturated (≥ 4 db) fatty acyl residues in GPC (Fig 3, S3 Fig) and GPE (S5 Fig) could also be verified in sperm of the carnivore (see also below). Lion sperm SM is again almost exclusively composed of saturated FA, but only sperm of bulls contain primarily long-chained unsaturated SM. Sperm of men, but also stallion and boar comprise both types of SM molecules.

Therefore, a characteristic pattern of PL types and FA residues was observed in each species and is only partly linked to the type of nutrition, although it has been reported that the FA composition of food determines individual FA in lipids to a certain degree [33]. For instance, addition of fish-oil to the diet can increase the n-3/n-6 ratio of PUFAs in boar sperm [34], but it is also described that a dietary increase in docosahexaenoic acid in sperm phospholipids is counterbalanced by a decrease in docosapentaenoic acid [35]. Moreover, only unsaturated FA that are endogenously present may be metabolically incorporated into boar sperm lipids *in vitro* [36].

Another hitherto unexpected species-specific feature concerns the occurrence of CL in SF of bulls, this lipid class having been described to be exclusively present in bacteria and mitochondria. However, contaminations by bacteria and mitochondria of defective sperm cells were excluded as principal source of CL by microscopic and microbiological examination.

### The functional requirement of sperm membrane fluidity is species-specifically implemented

It is generally accepted that the mammalian sperm head needs a fusogenic cell membrane to enable the final steps of fertilization (reviewed in [37]). This concerns (i) the acrosome reaction, an exocytotic event, which releases proteolytic enzymes necessary for sperm penetration through the zona pellucida, and (ii) the fusion of the sperm membrane with the oolemma. Moreover, a flexible tail membrane is a prerequisite for sperm motion. Therefore, the lipids of sperm membranes comprise high amounts of PUFA residues. These are parts of the GPC lipids, which mainly constitute both leaflets of the sperm cell membrane [38]. In addition, SM of the sperm head contain very-long carbon chains that are believed to be involved in acrosome reaction and membrane fusion during fertilization [39]. While in semen, ether-GPC mainly comprises polyunsaturated pentaenoyl or hexaenoyl residues in the *sn-2* position of the glycerol backbone, ester-GPC carries more saturated fatty acyl residues in that position. In animal species with a low amount of ether-GPC, a higher amount of ester-GPC including polyunsaturated molecules was found. Therefore, a functional threshold of unsaturation seems to be conserved for all mammalian sperm. However, human and lion sperm comprise a significant amount of less unsaturated ester-GPC, which consequently renders them less sensitive to oxidative stress. In contrast, plasmalogens are recognized as particularly vulnerable to oxidative stress because of their alkenyl ether linkage and, therefore, bull (ruminant) sperm are probably most sensitive to oxidative stress. They are followed at some distance by boar and stallion as well as human and lion sperm, where GPC plasmalogens are less abundant (boar, human, lion), or nearly completely missing (stallion), and the level of saturation is higher (human, lion).

### Accumulation of lyso-PL in sperm is largely prevented

An unexpected result of this study is the low abundance of LPL in sperm and SF of most animal species. In pig, horse and lion, we detected small amounts of (f)LPC only in semen from few single individuals. We can exclude a detection problem here, since LPC is detectable to the same extent as PC (or even more sensitively), as the head group (the same in both cases) determines the ionization efficiency. Furthermore, MALDI-TOF MS is a very sensitive method that allows the detection even in the order of femtomoles or even lower [40].

Higher but still moderate amounts of fLPC were detected in sperm from about half and in SF from all of the bovine individuals. This is in accordance with the suggested high susceptibility of bull sperm lipids to oxidation. Larger amounts of LPC exceeding the 5% level were only detected in human sperm and SF samples, although human sperm lipids may be considered less susceptible to oxidation according to the higher content of more saturated GPC.

The occurrence of LPL in ejaculates reflects a "snap-shot" after sperm maturation and storage in the male epididymis and the short-term exposure to SF directly after ejaculation (or catheter collection in the lion). We hypothesize that the accumulation of LPL is actually prevented in sperm until and during ejaculation. The original membrane lipids are effectively maintained and protected, since LPL acts as detergent and would thus disturb the membrane structure. In an experiment where boar sperm were artificially exposed to LPC, we could demonstrate that only 2% of incorporated LPC already had a negative impact on sperm motility and morphology (S8 Fig).

The reason for the considerable abundance of LPL in human ejaculates remains unknown - even more so since the occurrence of LPC was never related to lower sperm motility or poor morphology in the present study. Only in obese patients with a body mass index > 30, both a high incidence of LPC and a low sperm quality were previously observed [17]. Nevertheless, the comparatively large populations of morphologically abnormal sperm in human semen samples of the present study are likely to suffer from oxidative stress and contain or release significant amounts of LPL. Possibly, further lifestyle factors such as smoking, elevated scrotal temperature or pollutants like parabens, phthalate esters or bisphenols induce an irresistible oxidative stress in humans (reviewed in [41]). In contrast to humans, for the breeding animals (bulls, boars), the lifestyle is carefully controlled.

### Seminal fluid exerts sperm protection against ROS and supports repair of oxidized PL

LPL may only accumulate if lipid oxidation occurs and is accompanied by the removal of the oxidatively modified unsaturated fatty acyl residue (reviewed in [42]). This removal might happen by the action of PLA_2,_ although it is still unknown whether this enzyme preferentially targets oxidatively changed PL [43]. Both lipid oxidation and hydrolysis are timely and locally fine-tuned physiological processes in the course of fertilization. They are suggested to be crucial at the final stage of sperm transit in the female oviduct, but turn out to become detrimental if the physiological thresholds are exceeded too early and the mechanisms of lipid protection and repair are insufficient [23].

To understand how sperm lipids might be protected when sperm enter the female genital tract, we analyzed the capacity of SF to reduce radical compounds and thusminimize oxidative stress. We also analyzed the hydrolytic activity of SF, which might generate LPL from oxidized (but also from intact) PL, including ether-PL and plasmalogens [4, 44].

The hydrolytic activity was most pronounced in bovine and human SF and both species accumulate significant amounts of LPC in sperm and SF. However, no correlation exists between hydrolytic activity and LPC concentration across the individual samples within each species. This discrepancy can possibly be explained by a further hydrolysis of LPC into water-soluble products and/or its putative re-acetylation into GPC. The latter has been previously confirmed in boar sperm, where *de novo* synthesis of sperm lipids could be demonstrated. Externally offered FA were incorporated into sperm lipids and the sperm quality was sustained during long term storage [41, 42].

Interestingly, the rate of radical reduction was also most pronounced in bovine and human SF and an accumulation of (f)LPC was only possible in the individual samples with the lowest radical reduction capacity per sperm. Therefore, oxidation seems to be primarily responsible for the occurrence of LPL and enzymatic lipid hydrolysis is assumed to contribute in the second place to process oxidized PL. The radical reduction rate is also connected to the SF protein concentration, which again was most pronounced in bovine and human samples. The sulfhydryl, thioether and amino groups in proteins are effective scavengers of free radicals, and especially the cysteine thiols and their oxidized disulfide counterparts help to maintain the redox homeostasis in cells [45].

Conflicting negative relationships between overall protein concentration and sperm quality owed to the fact that SF comprises a huge variety of proteins, which not only provide their thiol and amino groups, but have ambivalent roles for sperm upon entrance in and transit through the female genital tract.

Since bull sperm are particularly susceptible to oxidative stress, the co-evolution of efficient protective mechanisms in semen might have been important. However, if the radical reduction rate, or the protein content is normalized to the sperm number in a given volume equivalent of SF, the protective capacity in bull semen is similar across the animal species. In contrast to that, the hydrolysis in bull SF remains much higher after normalization than in the other animal species. Actually, the powerful hydrolytic activity in bull SF could not solely be attributed to PLA_2,_ but also to the activity of a platelet-activating factor (PAF) acetylhydrolase [40]. This enzyme might preferentially hydrolyze the short-chained SL GPC analogues because of their structural similarity to PAF and could be essential not only in signaling events but also in the recycling of oxidized PL in bull semen.

In ungulates, particularly in the ruminants, a class of SF proteins, so-called fibronectin-like type II proteins, bind to the sperm cell membrane, stabilize it and create a protective envelope around the gamete (for review see [46]). According to their affinity for the GPC head group, these proteins could act as acceptors for sperm LPC. This would explain why more (f)LPC was found in SF compared to sperm and would support the idea that detrimental LPL need to be rapidly removed from the cells. Whether the PL that we detected in the organic extracts of SF samples contribute to sperm lipid metabolism, remains to be studied.

### Reproductive systems shape requirements for sperm protection

As stated in the previous paragraph, human SF provides a potent radical reduction capacity as well as hydrolytic activity. Particularly, if normalized to the number of sperm cells in the respective ejaculate volume, the values for both studied processes in human by far exceed the values revealed in the investigated animal species. In this context, we note that the human sperm concentration in general had decreased by more than 52.4% between 1973 and 2011 according to a study on a representative cohort of unselected men [44]. Despite the high activity of protecting enzymes, the occurrence of LPL in human is also exceptional in comparison to the animal species. Although human sperm PL are not considered as susceptible to oxidation as ungulate sperm lipids, human sperm and SF often contain a high quantity of LPC. It is not known to what extent the more (ungulates) or less (lion in captivity) relevant selection of the investigated individuals via long-term reproductive success upon breeding in comparison to the human individuals has an impact on the data of the study. Also age, health and lifestyle factors are surely more heterogeneously distributed among the human individuals of the study compared to the animals. Since we only have cumulative data on human individuals of this study, correlative research projects will be addressed in future.

A similarly small number of sperm for mating is provided by lion ejaculates, which are highly concentrated, but have the lowest volume across the investigated species. Lion sperm have the longest and most challenging way until reaching the site of fertilization in the oviduct because of their pre-vaginal deposition during mating. A similar situation applies for human and bull semen, which are also deposited into the anterior part of the female genital tract, the vagina (reviewed in [47, 48]). While human and lion sperm may be considered less susceptible to oxidation during their journey to fertilization, bull sperm are highly sensitive and need particular protection. We suggest that the high sperm concentration of bull ejaculates resulting in a high number of deposited sperm is an "investment" to compensate for the sperm sensitivity to oxidative stress. Boar and stallion sperm are deposited deep into the cervix or even into the uterus during mating [48–50]. The secretions of male accessory sex glands (in particular of seminal vesicles and bulbourethral glands) are of high volume and the sperm concentration in the ejaculates is low. The higher total number of boar sperm per ejaculate might be a tribute to the higher number of offspring and a longer ovulation period in pig compared to horse.

Interestingly, no capacity to reduce radical compounds was detectable in SF of stallion by the presented approach. However, we observed that the organic extraction of lipids from stallion SF was hindered by a stable lipid association to protein. We assume that stallion SF proteins efficiently remove LPL from sperm, since an accumulation of LPL is prevented in stallion sperm, although oxidation-sensitive PL are present and a hydrolytic activity was found. These findings support the theory that SF has a "sink" function for LPL, as stated above for the bovine. Further support comes from SF of men, which also contains much higher LPC concentrations than sperm from the same individual.

### PL composition in blood–according to its uniform function—is similar between species

The lipids of erythrocytes and BP are not congruent with the lipids of sperm and SF within each species, but are much more homogeneous between the different species compared to the semen. This could have been caused by the fact that the blood system is more consistent between the animal species, while the reproductive systems differ across mammals. Therefore, the more marked differentiation of the lipids in the sperm and SF across the species could be an adaptation to these different reproduction systems.

The only exceptional property of blood PL across species concerns the predominant, but unexplained abundance of SM in bovine erythrocytes. In general, erythrocyte GPC are dominated by ester-GPC with two double bonds whereas GPE species are dominated by ester-GPE with one double bond. Much more and diverse LPL were detected in blood than in semen, which could be indicative of an enhanced oxidative pressure in blood compared to semen until shortly after ejaculation. Otherwise, the effective avoidance of LPL accumulation in sperm cells seems extremely important to secure their final fertility at the end of transit through the female genital tract, whereas erythrocytes are continuously renewed. The expected influence of the nutritional characteristics of the species on fatty acyl residues of PL was also not reflected in blood, neither in the mass spectra of PL, nor in those of LPL.

## Conclusions

Despite many open questions, we conclude that the species-relatedness primarily determines the lipid composition of sperm. We showed that the general requirement of a certain membrane fusogeneity for fertilization is differently implemented in sperm of the five investigated mammalian species.

Compared to semen, the lipid composition of erythrocytes and BP is far more homogenous across the species. In accordance with the species-specific PL composition, protection mechanisms against oxidative stress effectively prevent an accumulation of LPL in semen samples from boar, stallion and lion. Lion sperm that need to travel a rather long way to the site of fertilization comprise moderately unsaturated ester-PL and predominantly saturated SM that are less susceptible to oxidation. In bull, particular oxidation-sensitive molecules such as plasmalogens and highly unsaturated SM are protected by a pronounced radical reduction capacity and hydrolytic activity of the SF. Although human sperm contain less susceptible PL and SF possesses a significant protective capacity, human sperm and SF contain the highest amounts of LPL with high individual variability. Presumably, lifestyle factors add to the oxidative stress level, which cannot be controlled by the native preventive portfolio.

Future studies will provide a comprehensive understanding of these imbalances and help to design counteractive measures.

## Supporting information

S1 Fig ESI spectra of lysophosphatidylcholine (LPC) fractions from boar, bull, stallion, lion and human samples. Lipid extracts were separated on a normal phase high performance thin-layer chromatography (HPTLC) plate with chloroform/ethanol/water/triethylamine (30:35:7:35, by vol.) as the mobile phase. Plates were air-dried and stained with primuline (Direct Yellow 59, Sigma-Aldrich, Taufkirchen, Germany) (50 mg/l dissolved in acetone/water 80:20, by vol.). Lipids were made visible under UV light and marked with a pencil. LPC fractions were directly analyzed by coupling a TLC plate reader to an ESI mass spectrometer. Mass spectra were recorded in the positive ion mode. For further details on ESI-IT MS see main text. For peak assignment, please see S1 Table.
(TIF)

S2 Fig ESI spectra of sphingomyelin (SM) fractions from boar, bull, stallion, lion and human samples. Lipid extracts were separated on a normal phase high performance thin-layer chromatography (HPTLC) plate with chloroform/ethanol/water/triethylamine (30:35:7:35, by vol.) as the mobile phase. Plates were air-dried and stained with primuline (Direct Yellow 59, Sigma-Aldrich, Taufkirchen, Germany) (50 mg/l dissolved in acetone/water 80:20, by vol.). Lipids were made visible under UV light and marked with a pencil. SM fractions were directly analyzed by coupling a TLC plate reader to an ESI mass spectrometer. Mass spectra were recorded in the positive ion mode. For further details on ESI-IT MS see main text. For peak assignment, please see S2 Table.
(TIF)

S3 Fig ESI spectra of phosphatidylcholine (PC) fractions from boar, bull, stallion, lion and human samples. Lipid extracts were separated on a normal phase high performance thin-layer chromatography (HPTLC) plate with chloroform/ethanol/water/triethylamine (30:35:7:35, by vol.) as the mobile phase. Plates were air-dried and stained with primuline (Direct Yellow 59, Sigma-Aldrich, Taufkirchen, Germany) (50 mg/l dissolved in acetone/water 80:20, by vol.). Lipids were made visible under UV light and marked with a pencil. PC fractions were directly analyzed by coupling a TLC plate reader to an ESI mass spectrometer. Mass spectra were recorded in the positive ion mode. For further details on ESI-IT MS see main text. For peak assignment, please see S3 Table.
(TIF)

S4 Fig ESI spectra of phosphatidylinositol (PI) fractions from boar, bull, stallion and human lipid samples. Lipid extracts were separated on a normal phase high performance thin-layer chromatography (HPTLC) plate with chloroform/ethanol/water/triethylamine (30:35:7:35, by vol.) as the mobile phase. Plates were air-dried and stained with primuline (Direct Yellow 59, Sigma-Aldrich, Taufkirchen, Germany) (50 mg/l dissolved in acetone/water 80:20, by vol.). Lipids were made visible under UV light and marked with a pencil. PI fractions were directly analyzed by coupling a TLC plate reader to an ESI mass spectrometer. Mass spectra were recorded in the negative ion mode. For further details on ESI-IT MS see main text. For peak assignment, please see S4 Table.
(TIF)

S5 Fig ESI spectra of phosphatidylethanolamine (PE) fractions from boar, bull and stallion samples. Lipid extracts were separated on a normal phase high performance thin-layer chromatography (HPTLC) plate with chloroform/ethanol/water/triethylamine (30:35:7:35, by vol.) as the mobile phase. Plates were air-dried and stained with primuline (Direct Yellow 59, Sigma-Aldrich, Taufkirchen, Germany) (50 mg/l dissolved in acetone/water 80:20, by vol.). Lipids were made visible under UV light and marked with a pencil. PE fractions were directly analyzed by coupling a TLC plate reader to an ESI mass spectrometer. Mass spectra were recorded in the negative ion mode. For further details on ESI-IT MS see main text. For peak assignment, please see S5 Table.
(TIF)

S6 Fig ESI spectra of phosphatidylethanolamine (PE) fractions from lion and human samples. Lipid extracts were separated on a normal phase high performance thin-layer chromatography (HPTLC) plate with chloroform/ethanol/water/triethylamine (30:35:7:35, by vol.) as the mobile phase. Plates were air-dried and stained with primuline (Direct Yellow 59, Sigma-Aldrich, Taufkirchen, Germany) (50 mg/l dissolved in acetone/water 80:20, by vol.). Lipids were made visible under UV light and marked with a pencil. PE fractions were directly analyzed by coupling a TLC plate reader to an ESI mass spectrometer. Mass spectra were recorded in the negative ion mode. For further details on ESI-IT MS see main text. For peak assignment, please see S5 Table.
(TIF)

S7 Fig Hydrolysis of selected seminal fluid samples over time. The plots of hydrolysis measurements from boar and stallion seminal fluid were fitted by a linear curve (f(x) = a + b×x) and the plots from bull, lion and human were fitted by an exponential growth to a maximum (f(x) = a×e-b×x). Due to these different courses of the hydrolysis reaction between the species, the absolute hydrolysis at a given time point (10 min) was used to compare the mean values of the investigated individuals between the species (see Table 2 of the main text).
(PDF)

S8 Fig Effect of artificial LPC on boar sperm. Beltsville Thawing Solution (BTS, Minitüb GmbH)-diluted boar semen (20 × 10^6^ sperm/ml) was mixed with 20 μM lysophosphatidylcholine (LPC 16:0, Avanti Polar Lipids®, No 855675C). After incubation at 38°C for 30 min, the ratios of total motility (blank boxes) and sperm with an intact acrosome (striped boxes) were analyzed. The lipid extract of washed sperm of this experiment was analyzed by MALDI-TOF MS and the ratio of LPC to total GPC was calculated (for details see Material and Methods of the main text). Incubation with 20 μM LPC led to 2.4 ± 3.6% inserted LPC in sperm cell membranes. Significant differences in total motility and the percentage of sperm with an intact acrosome between the incubation with 20 μM LPC and controls are marked by asterisks (*P* = 0.006 and 0.003, respectively, Wilcoxon signed-rank test, n = 11).
(PDF)

S9 Fig Original TLC pictures. Lipid extracts were separated on normal phase high performance thin-layer chromatography (HPTLC) plates with chloroform/ethanol/water/triethylamine (30:35:7:35, by vol.) as the mobile phase. Plates were air-dried and stained with primuline (Direct Yellow 59, Sigma-Aldrich, Taufkirchen, Germany) (50 mg/l dissolved in acetone/water 80:20, by vol.). BP–blood plasma, SF–seminal fluid, st.–lipid standard mixture made of LPC16:0, SM16:0, PC16:0/18:1, PA 16:0/18:1, PI 16:1/18:1, PE 16:0/18:1, PG 16:0/18:1 (bottom up).
(TIF)

S1 Table Assignment of signals detected in ESI spectra from lysophosphatidylcholine (LPC) spots. (DOCX)

S2 Table Assignment of signals detected in ESI spectra from sphingomyelin (SM) spots. n.a.—not assigned.
(DOCX)

S3 Table Assignment of signals detected in ESI spectra from phosphatidylcholine (PC) spots. (DOCX)

S4 Table Assignment of signals detected in ESI spectra from phosphatidylinositol (PI) spots. (DOCX)

S5 Table Assignment of signals detected in ESI spectra from phosphatidylethanolamine (PE) spots. (DOCX)
