# Supplementary figures and images for: Seminal lipid profiling and antioxidant capacity: A species comparison
Source: PLoS One. 2022 Mar 8;17(3):e0264675. doi: 10.1371/journal.pone.0264675 (PMC8903242; doi:10.1371/journal.pone.0264675)

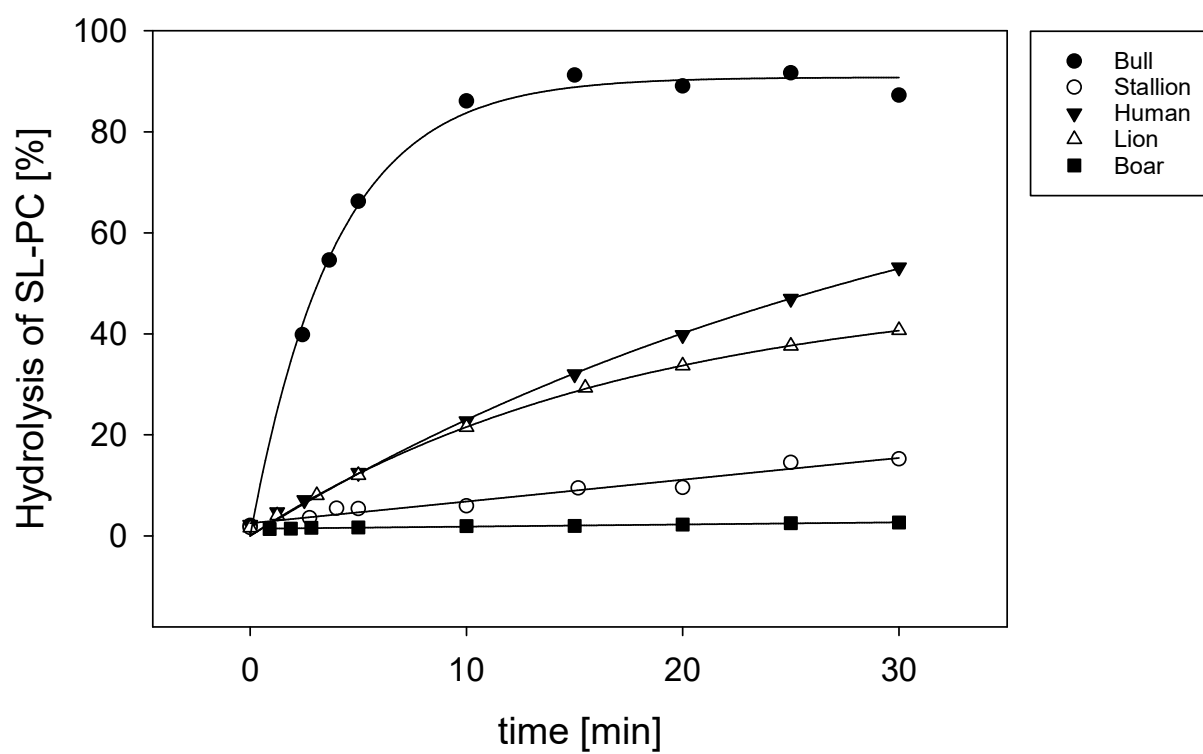

Supplement: S7 Fig — The plots of hydrolysis measurements from boar and stallion seminal fluid were fitted by a linear curve (f(x) = a + b×x) and the plots from bull, lion and human were fitted by an exponential growth to a maximum (f(x) = a×e-b×x). Due to these different courses of the hydrolysis reaction between the species, the absolute hydrolysis at a given time point (10 min) was used to compare the mean values of the investigated individuals between the species (see Table 2 of the main text). (PDF) [file pone.0264675.s007.pdf]

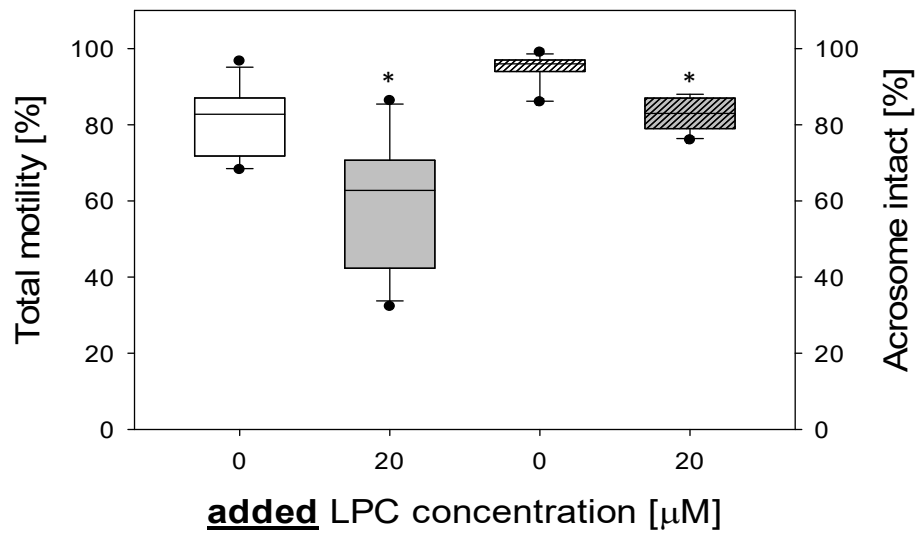

Supplement: S8 Fig — Beltsville Thawing Solution (BTS, Minitüb GmbH)-diluted boar semen (20 × 106 sperm/ml) was mixed with 20 μM lysophosphatidylcholine (LPC 16:0, Avanti Polar Lipids®, No 855675C). After incubation at 38°C for 30 min, the ratios of total motility (blank boxes) and sperm with an intact acrosome (striped boxes) were analyzed. The lipid extract of washed sperm of this experiment was analyzed by MALDI-TOF MS and the ratio of LPC to total GPC was calculated (for details see Material and Methods of the main text). Incubation with 20 μM LPC led to 2.4 ± 3.6% inserted LPC in sperm cell membranes. Significant differences in total motility and the percentage of sperm with an intact acrosome between the incubation with 20 μM LPC and controls are marked by asterisks (P = 0.006 and 0.003, respectively, Wilcoxon signed-rank test, n = 11). (PDF) [file pone.0264675.s008.pdf]

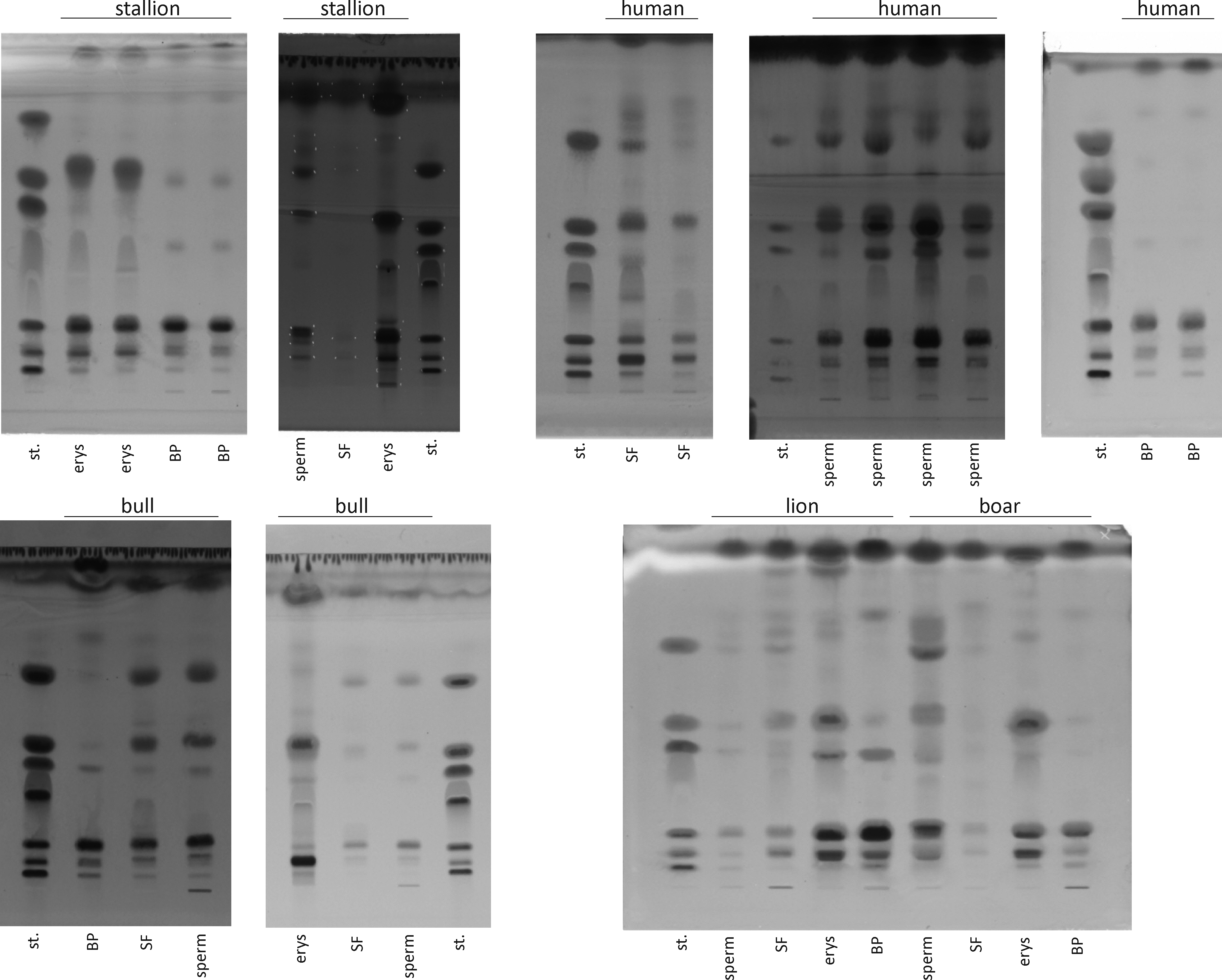

Supplement: S9 Fig — Lipid extracts were separated on normal phase high performance thin-layer chromatography (HPTLC) plates with chloroform/ethanol/water/triethylamine (30:35:7:35, by vol.) as the mobile phase. Plates were air-dried and stained with primuline (Direct Yellow 59, Sigma-Aldrich, Taufkirchen, Germany) (50 mg/l dissolved in acetone/water 80:20, by vol.). BP–blood plasma, SF–seminal fluid, st.–lipid standard mixture made of LPC16:0, SM16:0, PC16:0/18:1, PA 16:0/18:1, PI 16:1/18:1, PE 16:0/18:1, PG 16:0/18:1 (bottom up). (TIF) [file pone.0264675.s009.tif]
