# Supplementary material for: Seminal lipid profiling and antioxidant capacity: A species comparison
Source: PLoS One. 2022 Mar 8;17(3):e0264675. doi: 10.1371/journal.pone.0264675 (PMC8903242; doi:10.1371/journal.pone.0264675)
Supplement: S1 Table — (DOCX) [file pone.0264675.s010.docx]

**S1 Table.** Assignment of signals detected in ESI spectra from lysophosphatidylcholine (LPC) spots.

| ***m/z*** | **assignment** |
| --- | --- |
| 496.3 | [LPC16:0 + H]^+^ |
| 518.3 | [LPC16:0 + Na]^+^ |
| 524.3 | [LPC18:0 + H]^+^ |
| 540.3 | [LPC18:3 + Na]^+^ |
| 542.3 | [LPC18:2 + Na]^+^ |
| 544.3 | [LPC18:1 + Na]^+^ |
| 546.3 | [LPC18:0 + Na]^+^ |
| 566.3 | [LPC20:4 + Na]^+^ |
| 568.3 | [LPC22:6 + H]^+^ |
| 590.3 | [LPC22:6 + Na]^+^ |
