# Supplementary material for: Seminal lipid profiling and antioxidant capacity: A species comparison
Source: PLoS One. 2022 Mar 8;17(3):e0264675. doi: 10.1371/journal.pone.0264675 (PMC8903242; doi:10.1371/journal.pone.0264675)
Supplement: S2 Table — n.a.—not assigned. (DOCX) [file pone.0264675.s011.docx]

**S2 Table.** Assignment of signals detected in ESI spectra from sphingomyelin (SM) spots. n.a. - not assigned.

| ***m/z*** | **assignment** | ***m/z*** | **assignment** |
| --- | --- | --- | --- |
| 697.5 | [SM(d18:1/14:0) + Na]^+^ | 851.7 | [SM43:1;O2 + Na]^+^ |
| 703.6 | [SM(d18:1/16:0) + H]^+^ | 859.7 | [SM44:4;O2 + Na]^+^ |
| 705.6 | [SM(d18:0/16:0) + H]^+^ | 863.7 | [SM(d18:1/26:1) + Na]^+^ |
| 725.6 | [SM(d18:1/16:0) + Na]^+^ | 873.7 | [SM45:4;O2 + Na]^+^ |
| 727.6 | [SM(d18:0/16:0) + Na]^+^ | 875.7 | [SM45:3;O2 + Na]^+^ |
| 753.6 | [SM(d18:1/18:0) + Na]^+^ | 885.7 | [SM(d18:1/28:4) + Na]^+^ |
| 781.6 | [SM(d18:1/20:0) + Na]^+^ | 887.7 | [SM46:4;O2 + Na]^+^ |
| 807.6 | [SM(d18:1/22:1) + Na]^+^ | 901.7 | [SM47:4;O2 + Na]^+^ |
| 809.6 | [SM(d18:1/22:0) + Na]^+^ | 909.7 | [SM(d18:1/30:6) + Na]^+^ |
| 813.6 | [SM(d18:1/24:1) + H]^+^ | 913.7 | [SM(d18:1/30:4) + Na]^+^ |
| 821.6 | [SM41:2;O2 + Na]^+^ | 927.7 | [SM49:5;O2 + Na]^+^ |
| 823.6 | [SM41:1;O2 + Na]^+^ | 929.7 | [SM49:4;O2 + Na]^+^ |
| 835.6 | [SM(d18:1/24:1) + Na]^+^ | 937.7 | [SM(d18:1/32:6) + Na]^+^ |
| 837.6 | [SM(d18:1/24:0) + Na]^+^ | 965.7 | [SM(d18:1/34:6) + Na]^+^ |
