# Supplementary material for: Seminal lipid profiling and antioxidant capacity: A species comparison
Source: PLoS One. 2022 Mar 8;17(3):e0264675. doi: 10.1371/journal.pone.0264675 (PMC8903242; doi:10.1371/journal.pone.0264675)
Supplement: S3 Table — (DOCX) [file pone.0264675.s012.docx]

**S3 Table.** Assignment of signals detected in ESI spectra from phosphatidylcholine (PC) spots.

| ***m/z*** | **assignment** | ***m/z*** | **assignment** |
| --- | --- | --- | --- |
| 700.5 | [PC14:0/14:0 + Na]^+^ | 804.6 | [PC36:4 + Na]^+^ |
| 728.6 | [PC14:0/16:0 + Na]^+^ | 806.6 | [PC36:3 + Na]^+^ |
| 734.6 | [PC16:0/16:0 + H]^+^ | 808.6 | [PC36:2 + Na]^+^ |
| 746.6 | [PCo-16:0/18:1 + H]^+^ | 810.6 | [PC36:1 + Na]^+^  [PC38:4 + H]^+^ |
| 756.6 | [PC16:0/16:0 + Na]^+^ |  |  |
| 758.6 | [PC16:0/18:2 + H]^+^ | 812.6 | [PCp-16:0/22:6 + Na]^+^ |
| 760.6 | [PC16:0/18:1 + H]^+^ | 814.6 | [PCo-16:0/22:6 + Na]^+^ |
| 768.6 | [PCo-16:0/18:1 + Na]^+^ | 816.6 | [PCo-16:0/22:5 + Na]^+^ |
| 780.6 | [PC16:0/18:2 + Na]^+^ | 824.6 | [PCo-38:1 + Na]^+^ |
| 782.6 | [PC16:0/18:1 + Na]^+^ | 828.6 | [PC16:0/22:6 + Na]^+^ |
| 786.6 | [PC36:2 + H]^+^ | 830.6 | [PC16:0/22:5 + Na]^+^ |
| 790.6 | [PCp-16:0/22:6 + H]^+^ | 832.6 | [PC38:4 + Na]^+^ |
| 792.6 | [PCo-16:0/22:6 + H]^+^ | 840.6 | [PCp-18:0/22:6 + Na]^+^ |
| 794.6 | [PCo-16:0/22:5 + H]^+^ | 858.6 | [PC18:0/22:5 + Na]^+^ |
