# Supplementary material for: Seminal lipid profiling and antioxidant capacity: A species comparison
Source: PLoS One. 2022 Mar 8;17(3):e0264675. doi: 10.1371/journal.pone.0264675 (PMC8903242; doi:10.1371/journal.pone.0264675)
Supplement: S4 Table — (DOCX) [file pone.0264675.s013.docx]

**S4 Table.** Assignment of signals detected in ESI spectra from phosphatidylinositol (PI) spots.

| ***m/z*** | **assignment** | ***m/z*** | **assignment** |
| --- | --- | --- | --- |
| 809.6 | [PI16:0/16:0 – H]^+^ | 881.6 | [PI16:0/22:6 – H]^+^ |
| 835.6 | [PI16:0/18:1 – H]^+^ | 883.6 | [PI16:0/22:5 – H]^+^ |
| 837.6 | [PI34:0 – H]^+^ | 885.6 | [PI38:4 – H]^+^ |
| 857.6 | [PI36:4 – H]^+^ | 887.6 | [PI38:3 – H]^+^ |
| 861.6 | [PI36:2 – H]^+^ | 909.6 | [PI18:0/22:6 – H]^+^ |
| 863.6 | [PI36:1 – H]^+^ | 911.6 | [PI18:0/22:5 – H]^+^ |
| 865.6 | [PI36:0 – H]^+^ |  |  |
