# Supplementary material for: Seminal lipid profiling and antioxidant capacity: A species comparison
Source: PLoS One. 2022 Mar 8;17(3):e0264675. doi: 10.1371/journal.pone.0264675 (PMC8903242; doi:10.1371/journal.pone.0264675)
Supplement: S5 Table — (DOCX) [file pone.0264675.s014.docx]

**S5 Table.** Assignment of signals detected in ESI spectra from phosphatidylethanolamine (PE) spots.

| ***m/z*** | **assignment** | ***m/z*** | **assignment** |
| --- | --- | --- | --- |
| 696.5 | [PEo-34:4 ‒ H]^-^ | 740.5 | [PE36:3 ‒ H]^-^ |
| 698.5 | [PEo-34:3 ‒ H]^-^ | 742.5 | [PE36:2 ‒ H]^-^ |
| 700.5 | [PEo-16:0/18:2 ‒ H]^-^ | 744.5 | [PE36:1 ‒ H]^-^ |
| 702.5 | [PEo-16:0/18:1 ‒ H]^-^ | 746.5 | [PEp-16:0/22:6 ‒ H]^-^ |
| 714.5 | [PE16:0/18:2 ‒ H]^-^ | 748.5 | [PEo-16:0/22:6 ‒ H]^-^ |
| 716.5 | [PE16:0/18:1 ‒ H]^-^ | 750.5 | [PEo-16:0/22:5 ‒ H]^-^ |
| 718.5 | [PE34:0 ‒ H]^-^ | 760.5 | [PE16:1/22:6 – H]^-^  [PEo-16:0/22:6 ‒ H]^-^ |
| 722.5 | [PEo-16:0/20:5 ‒ H]^-^ |  |  |
| 724.5 | [PEo-16:0/20:4 ‒ H]^-^  [PE35:4 ‒ H]^-^ | 762.5 | [PE16:0/22:6 ‒ H]^-^ |
|  |  | 764.5 | [PE16:0/22:5 ‒ H]^-^ |
| 726.5 | [PEo-36:3 ‒ H]^-^  [PE35:3 ‒ H]^-^ | 766.5 | [PE38:4 ‒ H]^-^ |
|  |  | 768.5 | [PE38:3 ‒ H]^-^ |
| 728.5 | [PEo-36:2 ‒ H]^-^  [PE35:2 ‒ H]^-^ | 774.5 | [PEp-18:0/22:6 ‒ H]^-^ |
|  |  | 786.5 | [PE18:1/22:6 ‒ H]^-^ |
| 730.5 | [PEo-36:1 ‒ H]^-^  [PE35:1 ‒ H]^-^ | 790.5 | [PE18:0/22:6 ‒ H]^-^ |
|  |  | 792.5 | [PE18:0/22:5 ‒ H]^-^ |
| 738.5 | [PE16:0/20:4 ‒ H]^-^ | 794.5 | [PE40:4 ‒ H]^-^ |
